# Randomized Clinical Study on the Efficacy of Direct Anterior Approach Combined With Tendon Release and Repair After Total Hip Arthroplasty

**DOI:** 10.3389/fsurg.2022.845478

**Published:** 2022-03-21

**Authors:** Guanbao Li, Qiuan Chen, Wei Zhou, Pinquan Li, Peng Ma, Tongyuan Liu, Hai Tang

**Affiliations:** Department of Orthopaedics, The First Department of Hip Joint, Yulin Orthopedic Hospital of Chinese and Western Medicine, Yulin, China

**Keywords:** direct anterior approach (DAA), THA—total hip arthroplasty, conjoint tendon, joint capsule, repair

## Abstract

**Background:**

To study the effect of reconstruction of the joint capsule and conjoint tendon on the functional recovery of the hip joint during direct anterior approach (DAA) total hip arthroplasty.

**Methods:**

A total of 60 patients who underwent their first total hip arthroplasty surgery were selected. According to the set criteria, the selected patients were divided into observation group A (*n* = 30) and control group B (*n* = 30). In group A, the joint capsule and conjoint tendon (superior muscle, internal obturator muscle, and inferior muscle) were repaired *in situ*, while in group B, only the joint capsule was repaired *in situ*, and the conjoint tendon was not repaired. The surgical indicators, including hip joint function and clinical efficacy of the two groups, were compared.

**Results:**

After 6 months of follow-up in groups A and B, no dislocation occurred. The Harris Hip scores of group A were higher than those of group B at 1-month post-operation, i.e., *p* < 0.05, as well as the valid muscle strength and conjoint tendon valid tension, were higher in group A than group B at 1-month postoperative follow-up, i.e., *p* < 0.05.

**Conclusion:**

DAA for total hip arthroplasty on the premise of reconstructing the joint capsule structure can rebuild the tension of the conjoint tendon, enhance its muscle strength, and significantly improve the joint stability and function of the patient early stage. It is beneficial for the patient's rapid recovery and is worth implementing.

## Introduction

Total hip arthroplasty (THA) is an effective orthopedic operation that has evolved significantly in surgical methods and procedures since its inception (1–3). THA was initially reserved for the elderly and feeble patients with locomotor impairments and comorbidities (1). However, patients undergoing THA are increasingly opting for renowned, high-performance hip implants to meet their postoperative demands.

The posterolateral and mini posterior methods, the lateral approach, and the anterolateral approach are traditional surgical procedures for THA; the posterior approach is the most often utilized internationally and has survived the test of time (4–6). The disinsertion of the external rotators is the fundamental disadvantage of the posterior approach (PA), which is the most often utilized method in the United States and possibly worldwide (5, 7). Although newer studies have shown no higher risk when sufficient capsulorrhaphy or improved posterior soft tissue healing is achieved, posterior dislocation is possible for this method (8–11). Recently, there has been a trend toward minimally invasive surgical methods for a quick recovery; in this sense, orthopedic surgeons and patients worldwide have been paying close attention to the direct anterior approach (DAA).

The direct anterior approach (DAA) to the hip tends to have a longer shelf life in terms of popularity. The DAA employs a genuine internervous, intermuscular plane to expose the hip using the Hueter interval between the tensor fascia lata (TFL) and sartorius muscle. The strategy, according to proponents, is linked to reduced muscle injury and discomfort, as well as a faster recovery following hip arthroplasty. Despite the extensive research on the DAA and conventional techniques for primary THA, there is still disagreement over which technique is the most effective or desirable (6, 12).

However, exposing the femur can be challenging, and early users of the procedure have reported a high rate of early sequelae, including trochanteric and femoral fractures (13, 14). The conjoint tendon may need to be loosened during the DA approach to provide surgeons access to the hip joint. The conjoined external rotators tendon (CERT; conjoined tendons of the gemellus superior, obturator internus, and gemellus inferior) has been proposed as one of the critical active stabilizers of the hip, and it, along with the internally rotating gluteus minimus, is often referred to as the “rotator cuff” of the hip (15). The CERT can help with external hip rotation and hip joint stability (16), and it is also vital for avoiding posterior hip dislocation after THA (10, 17). Although the anterior or anterolateral minimally invasive method is touted as a muscle-sparing THA, surgeons may need to release more soft tissue for femur canal preparation, such as the capsular ligament or the insertion of muscles around the hip, including the CERT. These releases make it possible to properly prepare the canal, insert the broach, and test it.

Even though external rotation is thought to be significant for patient satisfaction (18), no investigation on repairing the conjoint tendon has been done. The purpose of this study was to investigate the effect of repairing the conjoint tendon on the postoperative hip joint function on the premise of reconstructing the joint capsule during the DAA for THA.

The objective of the study was to answer the following clinical question: Among patients with THA (*n* = 60), does the repairing of the conjoint tendon along with joint capsule, when compared with the repairing of joint capsule only, improve the Harris Hip Score (HHS) and manual muscle testing score (MMT), and decrease the frequency of postoperative complications, i.e., postoperative dislocation, infection, hematoma, and deep vein thrombosis?

The investigators hypothesized that repairing the conjoint tendon with a joint capsule improves the HHS and MMT scores and decreases the incidence of postoperative complications, i.e., postoperative dislocation, infection, hematoma, and deep vein thrombosis in the treatment of the control group.

## Materials and Methods

The study is a clinical randomized controlled trial comprising of patients who were selected for THA. The patients who visited the Department of Orthopedics from July 2020 to July 2021 were recruited for the present study. The present study was approved by the institute's ethical committee (Yulin Orthopedic Hospital of Chinese and Western Medicine) and was performed according to the Helsinki Declaration. Furthermore, the study's authors confirm that this trial has been registered (protocol YOHGU #IRB/2020/522), and all patients enrolled in the study have signed the written informed consent.

In this study, adult patients without any systemic complications, who strictly met the inclusion criteria, were engaged. The study's inclusion criteria were unilateral symptomatic hip osteoarthritis, Dorr's femur classification A/B, American Society of Anaesthesiologists Score (ASA) 3 or less, a body mass index (BMI) <40 kg/m^2^, and age between 40 and 80 years, the general condition of body is good, and there is no serious organic disease or infectious disease, total hip arthroplasty for the first time, piriformis muscle and external obturator muscle must be kept intact.

If a subject had a Dorr's femur classification of C, had previous hip surgery (excluding arthroscopy), refused to accept randomization and blinding, or had severe pathology that would affect postoperative participation, such as neurologic, psychiatric, or other confounding pre-existing musculoskeletal disorders, they were excluded from the study. In addition, those with cardiopulmonary insufficiency or cardiovascular and cerebrovascular illnesses, those with a history of trauma, tumor, or infection at the site, those who are contraindicated in surgery, those with insufficient follow-up data, and those who have insufficient follow-up data which are mentally ill were also excluded.

### Study Sampling

The patient sample size was calculated using (19):


(1) $$\begin{array}{l} n=\frac{N}{1+N{\left(e\right)}^{2}} \end{array}$$

Where *N* is the population size and *e* is the level of precision. For the present study, the population's size was determined based on the previous number of patients seen because of osteoarthritis, *N* is 60 patients for 6 months, and *e* is 0.05 at 95% confidence interval *n* = 60. The two groups received equal patients.

Irrespective of age and gender, randomization of consecutive patients was done using the concealed method. An independent researcher not involved in participant recruitment, treatment, or assessment prepared the randomization sequence with allocation prepared in sequentially numbered opaque envelopes. The surgeon and the primary investigator were blinded to the approach until the preoperative planning meeting, while participants were blinded pre-operatively. The patients were divided into group A (Experimental group, i.e., repairing the joint capsule and conjoint tendon) and group B (Control group, i.e., repairing the joint capsule only). A single surgeon who was an experienced senior surgeon had operated on all the patients under standard aseptic conditions and protocol to remove bias.

The artificial joint used in operation was all biologically fixed prostheses. Group A was sutured *in situ* on the loosened joint capsule and conjoint tendon (superior muscle, internal obturator muscle, inferior muscle). Meanwhile, only the joint capsule was sutured *in situ* in group B but not the conjoint tendon. The surgical indicators, including hip joint function and clinical efficacy of the two groups, were compared.

### Operative Procedure

**Figure 1:**
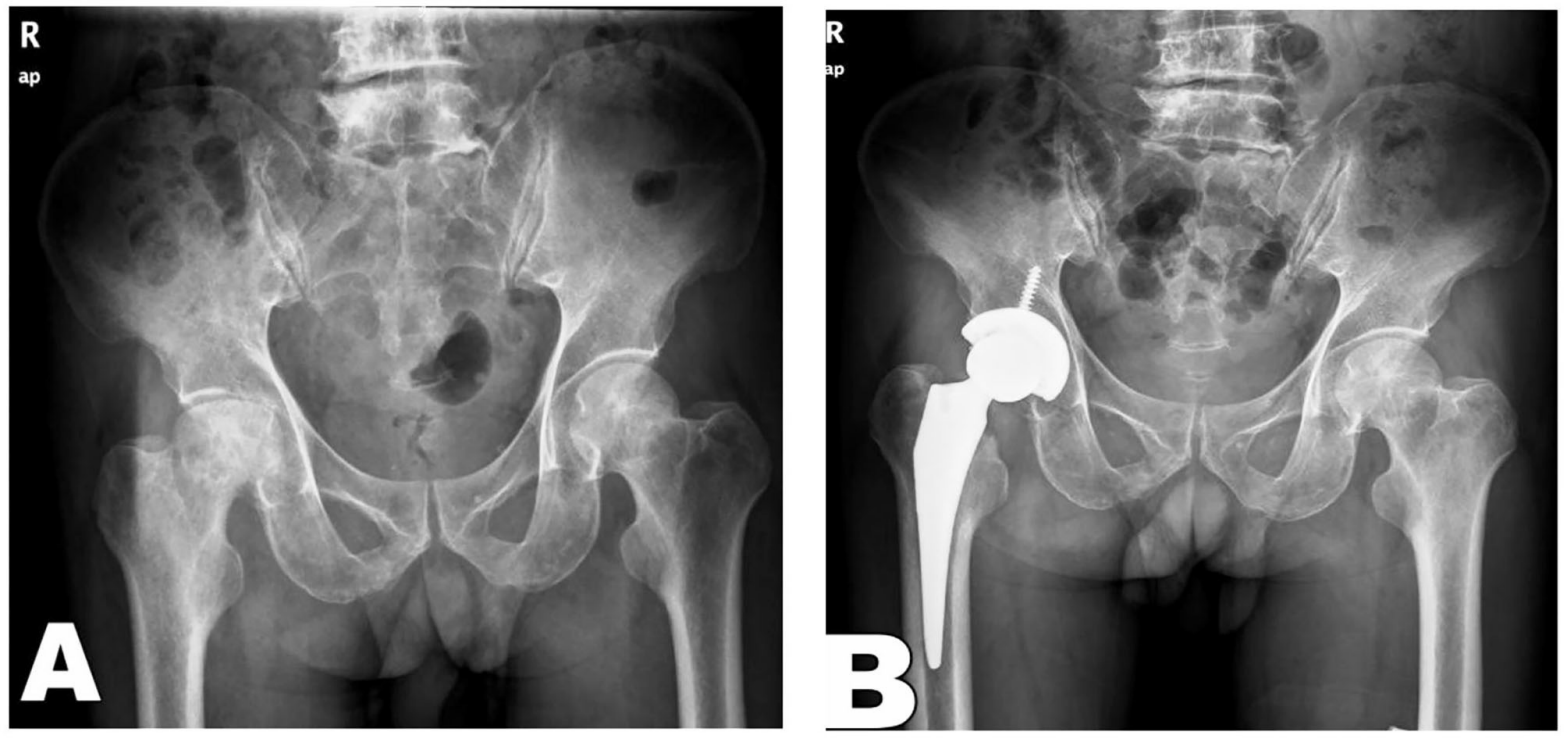
Preoperative X-rays indicated Ficat stage IV of hip avascular necrosis of the right femoral head and subjected to DAA for total hip arthroplasty. **(A)** Preoperative orthographic X-ray; **(B)** Postoperative orthographic X-ray.

**Figure 2:**
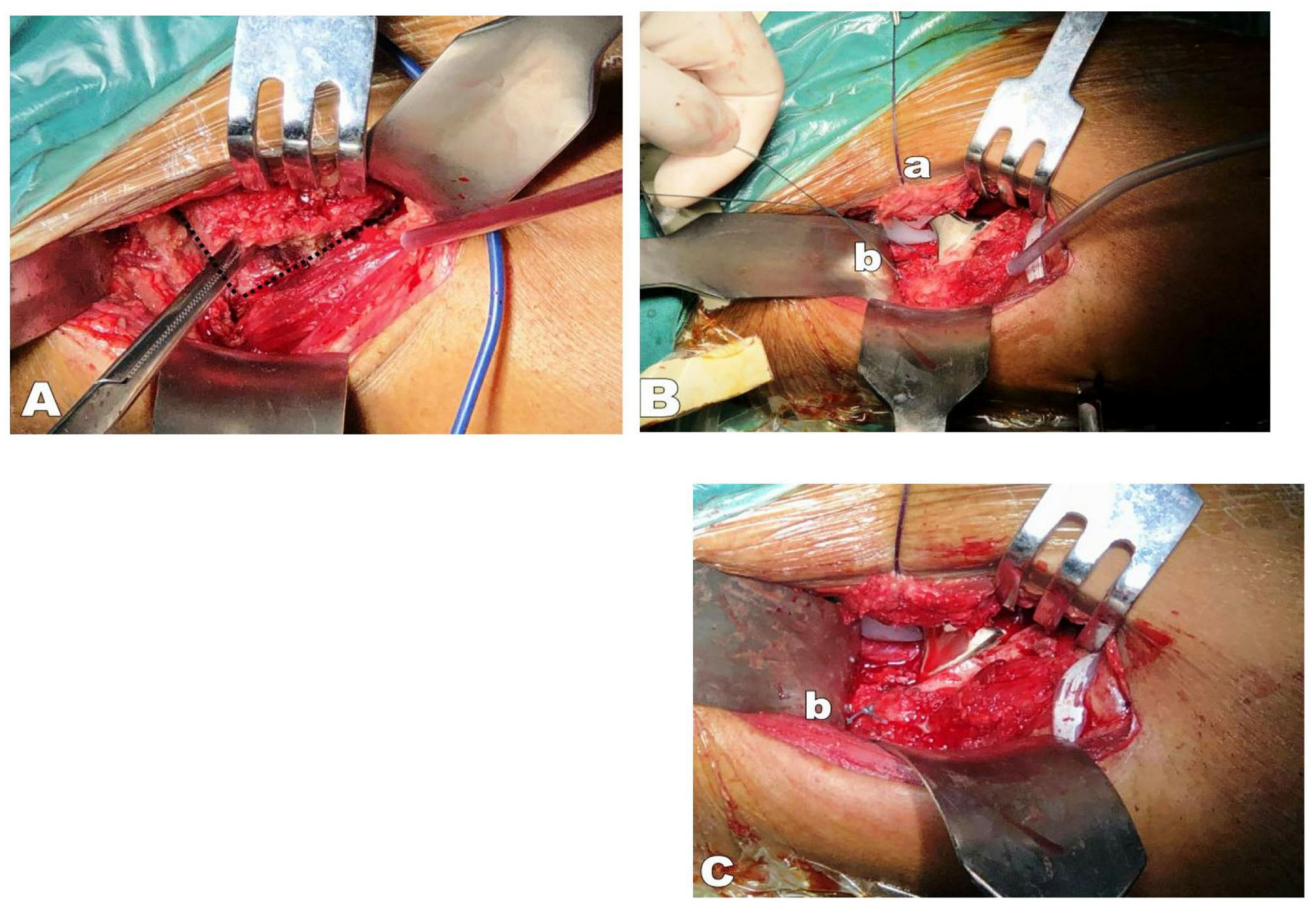
**(A)** The black dotted line indicates the cut from the upper edge of the femoral neck to the intertrochanteric line in an “L” shape, with a margin of 2–3 mm at the edge to facilitate the joint capsule reparation; **(B)** The loosened joint capsule was pulled (a) upward to expose the hip joint; Ethibond suture two was used to mark the conjoint tendon (b) in the proximal femur before it was cut off at the medial surface of the proximal femur of the greater trochanter (leave 2–3 mm at the stump of the greater trochanter to facilitate reconstruction); **(C)** The conjoint tendon was repaired *in-situ* after the prosthesis was installed (b); **(D)** Completed repair of the joint capsule (a).

Both group patients underwent intraspinal anesthesia in a supine position and with DAA incisions. The skin incision was 1 cm down from the anterior superior iliac spine and 2 cm behind the anterior superior iliac spine. Furthermore, the incision extended toward the head of the fibula following the direction of the tensor fascia lata muscle. Then, a peel hook was used to open and cut the fat layer on the fascia surface to make a blunt separation to the inside. After that, the ascending branch of the lateral femoral circumflex artery was ligated to expose the hip joint capsule through the gap of superficial Hueter between the lateral edge of the sartorius muscle and the anterior fascia of the tensor fascia lata muscle, followed by the gap of deeper Hueter between the lateral edge and the rectus femoris and the anterior edge of the gluteus medius. The joint capsule was cut from the upper edge of the femoral neck to the intertrochanteric line in an “L” shape, leaving the joint capsule opened, and absorbable thread was used to pull the joint capsule upwards to expose the joints and perform the artificial joint replacement. Group A: To obtain the optimal lifting height of the proximal femur during the operation and achieve a smooth operation of femoral medullary cavity shaping and prosthetic stem placement, Ethibond suture 2-0 was used to mark the conjoint tendon. It was cut off at the medial surface of the proximal femur of the greater trochanter (leave 2–3 mm at the stump of the greater trochanter) after the prosthesis was installed, and the conjoint tendon was sutured *in-situ* to close the entire incision site layer by layer after repairing the joint capsule according to the marked point. Group B: The conjoint tendon relaxation was performed with a similar method. After the prosthesis was installed, the conjoint tendon was not sutured. After the joint capsule was repaired, the entire incision was closed layer by layer. A drainage tube was placed in the two groups when the wound was sutured, and it was removed 24 h later (Figures 1, 2).

Both DAA groups utilized comparable intra-operative local infiltration anesthetic regimens based on a version of Kerr's technique. A 0.25% Bupivacaine, 20 mg ketorolac, and 1% adrenaline were used in the procedure.

Moreover, the individuals with symptoms of renal failure were not administered ketorolac. For the first 24 h following surgery, continuous infusion pumps were utilized on the ward. All patients received prophylactic antibiotics (Ceftriaxone) and thromboprophylaxis, as required by the hospital service.

All of the patients were able to move about the day after surgery. The DAA group's hip mobility was unaffected. The goal for discharge to home or rehabilitation was set for the 3rd postoperative day. On a daily basis, physiotherapists and physicians accompanying the orthopedic team monitored this. Patients who did not meet the criteria for release were sent to a rehabilitation facility.

### Outcome Variables

Participants were assessed pre-operatively and 2 weeks, 1, 3, and 6 months after THA. The same investigator was in charge of the follow-up.

The Harris Hip Score (HHS) was used to evaluate the hip joint function before and after the operation, divided into four aspects: pain, function, deformity, and joint mobility. The total score is 100 points; a score ≥90 is excellent, 89–80 is good, 79–70 is fair, and a score lower than 70 is poor.

The manual muscle test (MMT) was used to detect the abductor muscle strength of the two groups of patients. The evaluation method was divided into six grades, and the valid muscle strength is not lower than the 3rd grade.

**Figure 3:**
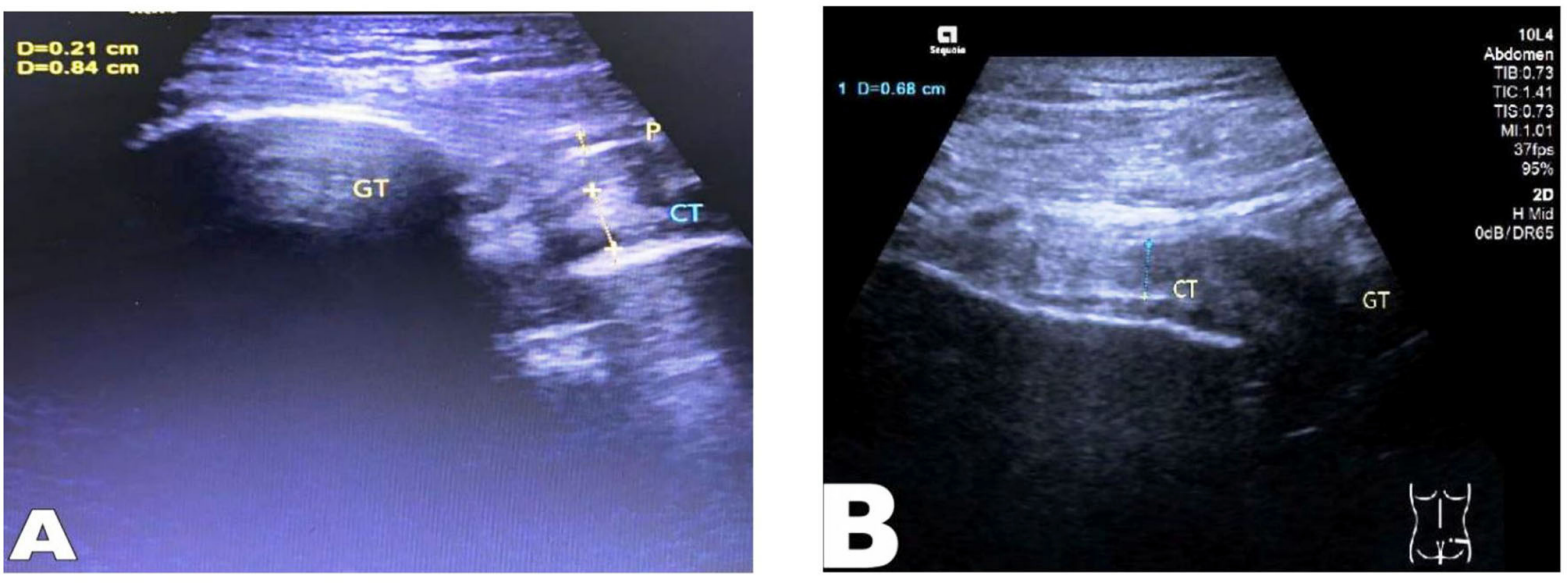
Musculoskeletal ultrasound examination, **(A)** “CT” indicates the preoperative conjoint tendon morphology; “GT” indicates the greater trochanter of femur; **(B)** “CT” indicates the conjoint tendon morphology after repaired, with good tendon tension.

**Figure 4:**
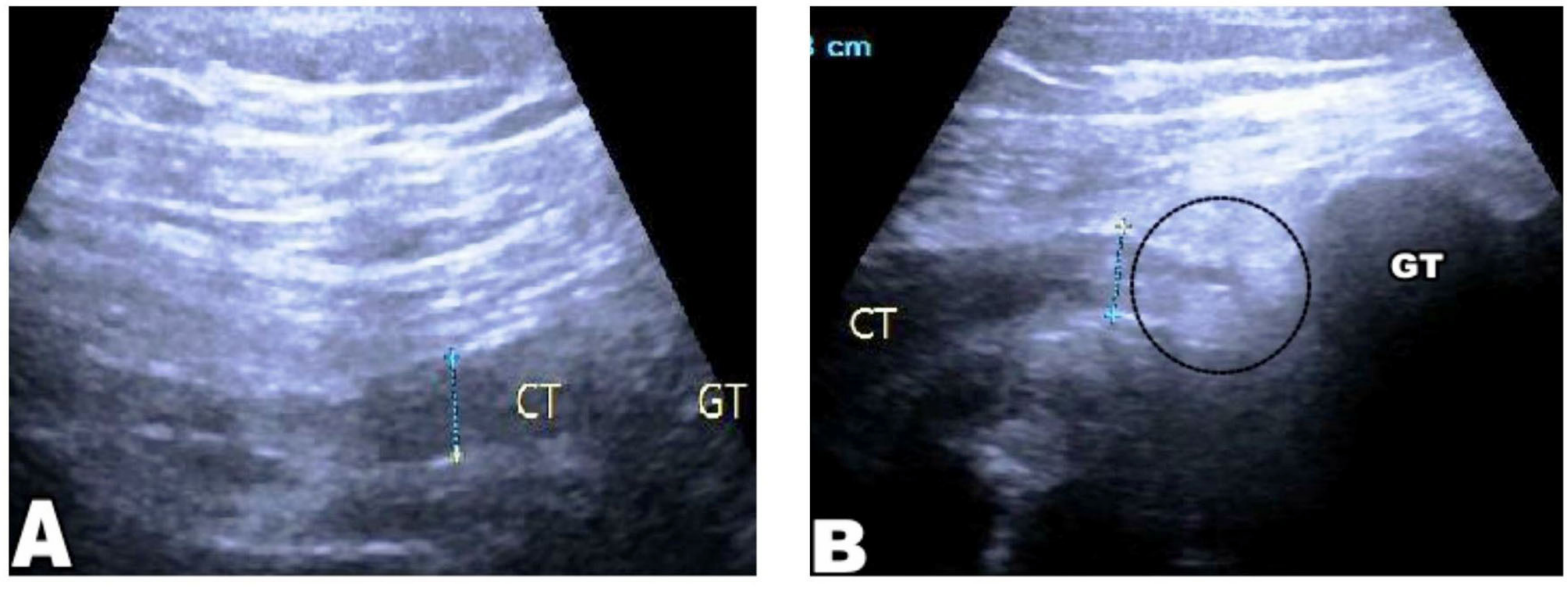
Musculoskeletal ultrasound examination, **(A)** “CT” indicates the preoperative conjoint tendon morphology; “GT” indicates the greater trochanter of the femur; **(B)** “CT” indicates the conjoint tendon morphology after lysis; the continuity of the tendon is interrupted, slightly tortuous, and has poor tension. The black circle indicates the position of the interrupted conjoint tendon.

The tension of the conjoint tendon of the two groups was compared by musculoskeletal ultrasound examination. A good continuity without tortuosity in a conjoint tendon represents valid tension. However, a discontinuous conjoint tendon with tortuosity, shortening, or unclear display represents invalid tension (Figures 3, 4).

The operation time, intra-operative and postoperative total blood loss, and related complications of the two groups of patients were recorded.

### Statistical Analysis

SPSS 22.0 (SPSS, Inc., Chicago, IL) statistical software was used to analyze the data. Measurement data were expressed as mean ± standard deviation (χ^2^ ± S) and paired *t*-test was used to compare groups. Count data is expressed as an example (percentage), and the comparison between groups uses the χ^2^-test. Statistical significance was assigned to findings with *p*-values of <0.05.

## Results

**Figure 5:**
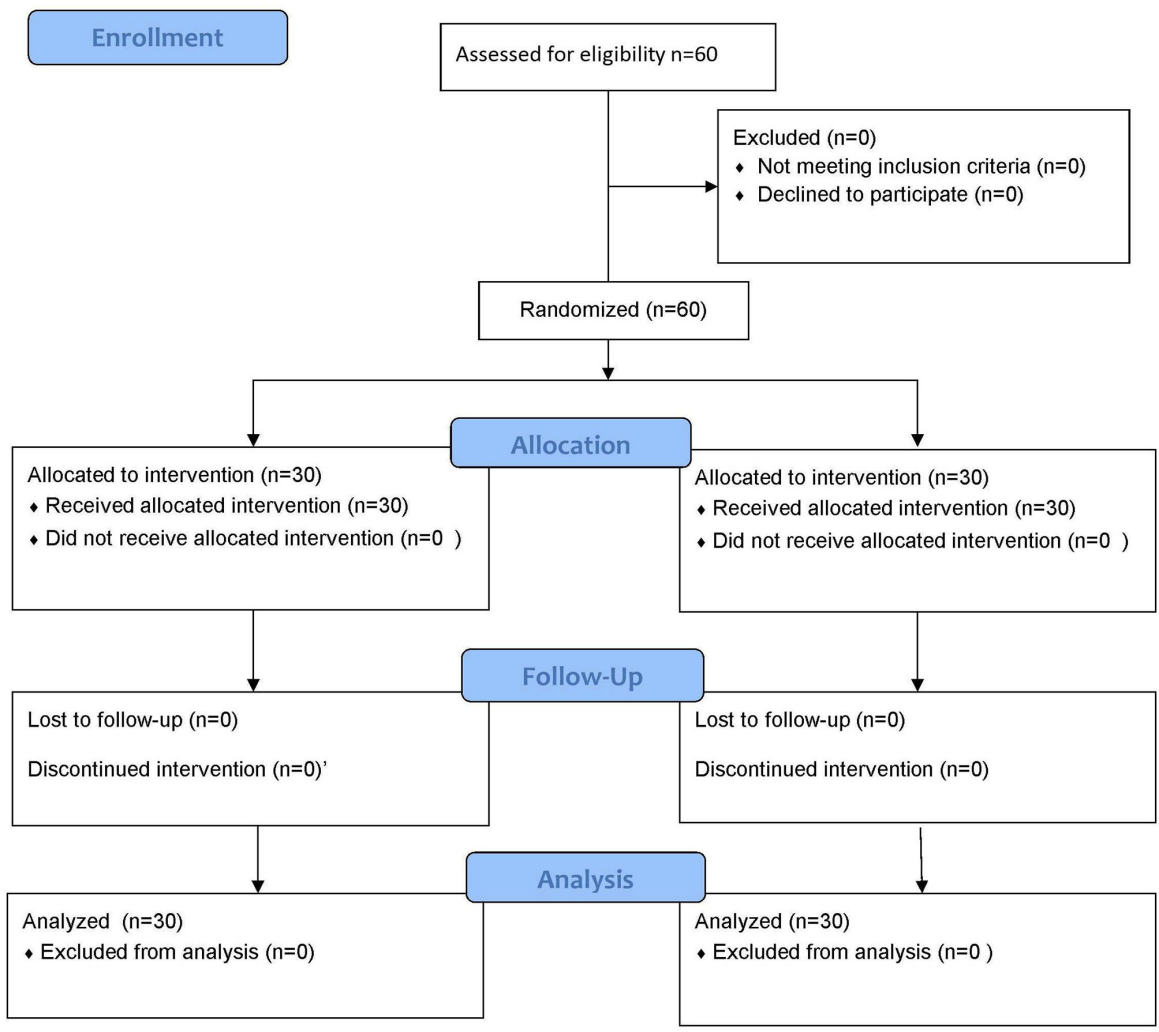
Demonstrates the flow diagram for patient recruitment and selection.

Group A comprised 18 males and 12 females aged 45–81 years old, average (64.7 ± 3.1) years old. Meanwhile, Group B consisted of 16 males and 14 females ranging from 48 to 80 years old, average (64.5 ± 3.0) years old. Comparison of general data such as gender, age, and disease diagnosis between the two groups of patients was not statistically significant (*p* > 0.05), and they were comparable. Figure 5 demonstrates the flow diagram for patient recruitment and selection.

There was no significant difference in operation time and blood loss between the two groups (*p* > 0.05) (Table 1).

**Table 1:** Comparison of surgical indicators between the two groups (χ^2^ ± S).

**Groups**	**Intraoperative blood loss (ml)**	**Operation time (min)**	**Incision length (cm)**	**Postoperative drainage volume (ml)**
A	180.4 ± 35.9	63.1 ± 15.4	9.34 ± 2.0	180.8 ± 31.7
B	179.7 ± 35.5	61.8 ± 13.9	9.35 ± 1.8	196.7 ± 22.2
*t*-value	0.550	0.576	0.250	8.788
*P*-value	>0.05	>0.05	>0.05	<0.05

One month after the surgery, the Harris Hip score of group A was higher than that of group B, and the difference between the groups was statistically significant (*p* = 0.012, *p* < 0.05). Furthermore, three to 6 months after the surgery, the HHS of the two groups was not statistically significant (*p* = 0.23, *p* > 0.05) (Table 2).

**Table 2:** Comparison of Harris Hip scores between the two groups pre- and post-operation (χ^2^ ± S, score/min).

**Groups**	**Pre-operation**	**1-month post-operation**	**3–6 months post-operation**
A	45.7 ± 7.0	85.3 ± 11.3	90.2 ± 2.4
B	45.1 ± 7.2	80.4 ± 11.7	90.4 ± 2.9
*t*-value	0.33	2.35	0.27
*P*-value	>0.05	*p* = 0.012; *p* < 0.05	*p* = 0.23; *p* > 0.05

The valid muscle strength of group A was significantly higher than that of group B, and the difference was statistically significant (*p* = 0.045, *p* < 0.05). In addition, the valid tension of the conjoint tendon was significantly higher in group A than in group B, and the difference was statistically significant (*p* = 0.042, *p* < 0.05). However, there was no significant difference in the incidence of complications such as postoperative dislocation, infection, hematoma, and deep vein thrombosis between the two groups (*p* > 0.05) (Table 3).

**Table 3:** Valid muscle strength, conjoint tendon valid tension, and post-operative complications.

**Groups**	**Joint dislocation**	**Infection**	**Hematoma**	**Deep vein thrombosis**	**Valid muscle strength**	**Conjoint tendon valid tension**
Observation group (*n* = 30)	0 (0)	0 (0)	1 (3.33)	1 (3.33)	28 (82.50)	26 (86.7)
Control group (*n* = 30)	0 (0)	1 (3.33)	2 (6.67)	1 (3.33)	23 (62.50)	21 (70.0)
χ^2^-value	0.0	0.556	0.346	0.721	4.013	4.011
*P*-value	0.0	0.456	0.556	0.396	0.045	0.042

## Discussion

The main objective behind this study is to examine the effect of repairing of conjoint tendon and join capsule vs. repairing of joint capsule only; on the improvement of HHS and MMT scores and the incidence of postoperative complications, i.e., postoperative dislocation, infection, hematoma, and deep vein thrombosis. The results of this research confirm the hypothesis, i.e., a significant difference exists among group A and group B in terms of HHS and MMT scores (*p* < 0.05), i.e., group A shows a significant improvement in the HHS and MMT score at 1-month follow-up and reduced incidence of postoperative complications, i.e., postoperative dislocation, infection, hematoma, and deep vein thrombosis as compared to group B. However, at 3- and 6-month follow-up, the difference between groups A and B in terms of HHS and MMT is insignificant, i.e., *p* > 0.05.

Early dislocation is a severe complication after the first total hip arthroplasty, and this incidence is preceded only by the aseptic loosening of the joint prosthesis. Surgical factors are considered to be one of the critical factors affecting the incidence of early dislocation, and they are also the most concerned the researchers. Surgical factors include prosthetic factors and surgical operating factors, while surgical operating factors include replacement approach, soft tissue imbalance reconstruction, the design and placement of the prosthesis, patient compliance, history of previous hip surgery. Dislocations usually occur within 3 months after the replacement, and late dislocations are predominant (20). With the development of hip arthroplasty and the maturity of the replacement experience of the surgeon, the dislocation issue caused by the poor positioning of the prosthesis is gradually reduced, and the soft tissue imbalance has gradually become the main factor of the dislocation of the prosthesis. Studies have shown (21) that the soft tissue around the hip has an important influence on the stability of the prosthesis.

The stability of the hip joint after the conventional total hip arthroplasty through the posterolateral approach is partly maintained by the tension of the hip girdle muscle and the repair of the fibrous scar around the prosthesis. However, the early postoperative joint prosthesis lacks the protection of the joint capsule. The antagonistic muscle strength balance between the external and internal obturator muscles (the anterior fiber of the gluteus medius and minimus) is more prone to posterior hip dislocation. Therefore, the patient must be required to strictly control the range of motion of the affected hip to prevent hip dislocation. Therefore, retaining the joint capsule and the anatomical structure of the small supinator muscle group during the process of total hip arthroplasty will help to restore the soft tissue balance of the hip joint and increase the joint stability (22). Wu et al. (23) believe that since soft tissue acts as an essential stable structure of the hip joint, it is crucial to restoring the balance of the soft tissue around the hip to maintain the stability of the hip joint after the replacement. Lu et al. (24) believe that low soft tissue tension, especially the abductor muscle weakness, is the most crucial cause of total hip arthroplasty dislocation. Hideki et al. (25) repaired the external obturator muscle before soft tissue enhancement can reduce the risk of prosthesis dislocation during the posterior total hip arthroplasty.

White et al. (26) performed total hip arthroplasty *via* the posterior approach. During the operation, the posterior joint capsule was trimmed into a tissue flap with a 30–50% circumference of the acetabular. After the prosthesis was implanted, the joint capsule and the external obturators were sutured within the same layer onto the 2.7 mm diameter bone hole on the greater trochanter of the femur. Six-month follow-up showed three out of 437 cases (0.7%) had a post-traumatic dislocation, and another 4 had asymptomatic avulsion fractures in the greater trochanter. The dislocation rate was as high as 4.8% in the other patients who did not undergo this repair. Sioen et al. (27) have performed posterior total hip arthroplasty on both hips of 3 fresh cadavers to observe the stability of the hip joint using different repair methods on the posterior joint capsule (no repair, soft tissue repair, muscle, and bone repair). Research suggests that the bone repair of the composite tissue flap of the posterior joint capsule and external obturator muscle group can significantly increase the stability of the hip joint. Zhang et al. (28) reported that using suture anchors to anchor the piriformis and external obturator muscle to the greater trochanter can reduce the dislocation. Browne et al. (29) reported a repair method in which the posterior joint capsule and short external obturator compound tissue flaps were directly sutured on the posterior edge of the gluteus minimus and its deep anterior and superior joint capsule. This method involves joint capsule repair and soft tissue end-to-end suture eliminating the dead space and significantly reducing early dislocation incidence.

Researchers have confirmed the vital role of repairing the joint capsule and short external rotators in the THA of the posterolateral approach to maintain the early stability of the hip joint. In recent years, with the continuous development of minimally invasive techniques, there have been more methods of the THA surgical approach. Among them, the direct anterior approach (DAA) is a new minimally invasive surgical technique that is currently widely available and clinically applied in total hip arthroplasty (30). This technology uses the muscle gap between the tensor fascia lata and sartorius muscles as well as the rectus and gluteus medius muscles to expose the hip joint, hence, avoiding damage to the abductor muscles around the hip joint and ensuring the integrity of the soft tissues on the back of the hip joint. Hence, people named it the “Hueter” approach (31). Most studies have shown that this method has a lower incidence of complications such as minimal damage to muscle, mild pain, and dislocation and can enable patients to undergo total hip arthroplasty surgery and recover more quickly after the surgery. Therefore, it is widely used in clinical orthopedic applications.

Although the DAA has apparent advantages, it has limitations too. This approach enters the hip joint through the muscle gap. Since the intraoperative incision is small, the surgical field and operation space are narrow; thus, the femoral treatment is more complicated. In addition, if there is a lack of traction bed, unique prosthesis, operating tools, or immature surgical technique, complications such as iatrogenic fractures, nerve and muscle damage often occur during the operation. Therefore, the conventional DAA approach requires the hospital to be equipped with a traction bed, unique prosthesis, and operating tools.

Last but not least, strict screening is needed in every case. For instance, patients with high BMI indexes and obesity will be excluded. This method also increases the cost and difficulty of the surgery and the financial burden of patients. Fujii et al. have confirmed that relaxation of the conjoint tendon (internal obturator muscle, superior and inferior muscles) helps achieve the optimal lifting height of the proximal femoral (25) and ease the operation of the femoral medullary cavity shaping and prosthetic stem implantation. It will significantly reduce the surgical cost and difficulty of the DAA approach, which is conducive to promoting the DAA-THA approach. We have known that suturing the external obturator muscles in the posterolateral THA and repairing the posterior joint capsule can effectively provide strong support for the fragile posterior structure of the hip after THA. It will reduce postoperative joint dislocation (32, 33).

On the other hand, from the perspective of biomechanics, suturing the external obturator muscles and repairing the posterior joint capsule can promote the artificial hip joint of the body to be closer to the physiological state of the human body, thereby achieving an excellent soft-tissue balance, which is beneficial to the functional recovery of the hip joint of patients in the later stage. Based on this, we also found that in the DAA-THA approach, by comparing group A with both joint capsule and conjoint tendon repairs and group B with only joint capsule repair, the drainage rate of group A was significantly less than that of group A group B. Furthermore, the Harris score of group A was higher than that of group B 1-month post-operation. In addition, the valid muscle strength of group A was significantly higher than that of group B. Also, the musculoskeletal ultrasound showed that the valid tension of the conjoint tendon in group A was significantly higher than that in group B. Our research confirms that repairing the conjoint tendon on the premise of reconstructing the joint capsule during THA surgery through the DAA approach can indeed reduce postoperative complications and restore hip joint function faster.

Analyzing the reasons, we believe that it may be related to the following: Firstly, From the histological Analysis, reconstruction of the joint capsule and conjoint tendon can compensate for the weakness of the front joint structure after total hip arthroplasty, thereby effectively reducing the incidence of postoperative dislocation; In addition, from biomechanical Analysis, reconstruction of the joint capsule and conjoint tendon can bring the hip joint closer to the physiological state, thereby obtain a better soft tissue balance, and better restoring the joint function after the replacement.

## Limitations

The minimal number of patients included in the study is one of the study's shortcomings. On the other hand, most studies on long-term results after orthopedic surgeries have utilized a similar sample size. Patients were not evenly distributed in terms of sex between the two groups, and neither had a preoperative VAS score. Furthermore, the trial's follow-up was not designed to record the precise day when gait assistance was stopped. Observing bias might develop when data is gathered and analyzed by a single investigator.

In summary, THA surgery through the DAA approach to repair the conjoint tendon on the premise of reconstructing the joint capsule structure can rebuild its tension, enhance its muscle strength, and improve the patient's hip joint function, with a definite effect. Furthermore, combined with the advantages of the DAA approach, it can significantly reduce complications such as dislocation, bleeding, and infection after total hip arthroplasty, and it is worthy of clinical promotion.

## Data Availability Statement

The raw data supporting the conclusions of this article will be made available by the authors, without undue reservation.

## Ethics Statement

All procedures performed in studies involving human participants were in accordance with the ethical standards of the institutional and/or national research committee and with the 1964 Helsinki declaration and its later amendments or comparable ethical standards. This study protocol was reviewed and approved by [Yulin Orthopedic Hospital of Chinese and Western Medicine, Guangxi University], approval number [YOHGU #IRB/2020/522]. The patients/participants provided their written informed consent to participate in this study.

## Author Contributions

GL and QC: concept and designed the study. WZ: analyzed data. PL and PM: collected the data and helped in data analysis. TL and HT: drafting the manuscript. All authors contributed to the article and approved the submitted version.

## Conflict of Interest

The authors declare that the research was conducted in the absence of any commercial or financial relationships that could be construed as a potential conflict of interest.

## Publisher's Note

All claims expressed in this article are solely those of the authors and do not necessarily represent those of their affiliated organizations, or those of the publisher, the editors and the reviewers. Any product that may be evaluated in this article, or claim that may be made by its manufacturer, is not guaranteed or endorsed by the publisher.
